# Sexual and Reproductive Health Care for Women with Intellectual Disabilities: A Primary Care Perspective

**DOI:** 10.1155/2013/642472

**Published:** 2013-12-12

**Authors:** Nechama W. Greenwood, Joanne Wilkinson

**Affiliations:** ^1^Department of Family Medicine, Boston University School of Medicine, Dowling 5, 771 Albany Street, Boston, MA 02118, USA; ^2^Department of Community Health Sciences, Boston University School of Public Health, 801 Massachusetts Avenue, 4th floor, Boston, MA 02118, USA

## Abstract

Adults with intellectual disabilities (ID) face multiple health disparities and challenges to accessing health care. Little is known about sexual health care of this population and about how to optimize women's reproductive health care for women with intellectual disabilities. Women with ID face important barriers to care, including lack of provider training and experience, hesitancy to broach the topic of sexual health, a lack of sexual knowledge and limited opportunities for sex education, disability-related barriers, higher prevalence of sexual abuse and assault, often underreported, lack of dialogue around this population's human right to consensual sexual expression, undertreatment of menstrual disorders, and legal and systemic barriers. We conducted a limited literature review related to six aspects of sexual health care of women with ID, including barriers to sexual health care, sex education, sexual abuse and consensual sexuality, contraception, screening for sexually transmitted infections and cervical cancer, and pregnancy and parenting. After providing background information about each topic, we suggest practice recommendations for primary care clinicians, using a rights-based framework.

## 1. Introduction

Intellectual disability (ID, formerly mental retardation) is characterized by significant limitations in intellectual functioning (generally measured as IQ of 70–75 or less) and in adaptive behavior, including conceptual, social, and practical skills, that originates before the age of 18 [1]. Adults with intellectual disability (ID) face significant health disparities [2], including disparities in primary health care access [3, 4] cancer screenings and preventive health care access [5–9], health education uptake [10], mental health care and substance abuse treatment access [11, 12], and oral health [13–15]. There are also significant disparities in research participation [16, 17]^,^ which contributes to important gaps in knowledge about the health of this population. Reproductive and sexual health of women with ID is especially overlooked and understudied. The literature that does exist is often from the perspective of support workers and family members [18–23]. Recently, there has been limited scholarship focused on the perspectives and preferences of adults with ID [24–27]; however, important gaps in the literature remain. Though many adults with ID receive health care from primary care physicians [3, 28, 29], there is also a specific lack of primary care-focused research and practice guidelines for the sexual health care of adult women with intellectual disabilities.

In order to address these gaps, we conducted a limited review of the literature related to the sexual health care of women with intellectual disabilities. Though sexual health care is a very wide field, we choose to focus our review on six topics pertinent to primary care, including barriers to care, sex education, sexual abuse and consensual sexuality, contraception, screening for cervical cancer and sexually transmitted infections (STI), and pregnancy and parenting. Our major findings are summarized in Supplementary Table 1 available online at http://dx.doi.org/10.1155/2013/642472. Our literature review was conducted using PubMed and Google Scholar, and search terms included combinations of the following: intellectual disability, mental retardation, developmental disability AND sexual health, primary care, reproductive health, women's health, sex education, sexual health education, sexual abuse, sexuality, cervical cancer, cytology, sexually transmitted infection, sexually transmitted disease, pregnancy, prenatal, parent, parenting, and mothers. We excluded papers in languages other than English, a potential limitation of this review. We were interested in best practices for primary care and geared our search towards clinical literature. We will discuss clinical recommendations in each of these focus areas, preceded by a review of the literature. An important limitation of this paper relates to the lack of evidence and clinical guidelines for best practice; for each topic area we have noted which recommendations are evidence-based and which are theoretical- or postulated-based on empirical experience. Though recommendations are intended for use by individual primary care providers (i.e., family physicians, internists, nurse practitioners, etc.) who work in community settings, they may also be applicable to practitioners in other settings, such as institutional environments and care teams.

## 2. Barriers to Sexual Health Care in Primary Care Settings

### 2.1. Background

Prior to the 1970s, the majority of individuals with ID in the United States, [30], Australia [26], and the United Kingdom lived in institutions, where they also received any needed medical care, often from providers who care exclusively for these institutionalized adults. The shift from institutionalization to community living has had overall positive effects on behavior [31] and quality of life [32] for people with ID. Currently, most people with ID receive their primary care in community settings, from providers who care for the general public [33]. However, primary care education may not have caught up with the societal shift represented by deinstitutionalization [33, 34]. Primary care providers receive very little to no formal education in caring for this population [35, 36]. This lack of education creates barriers to effective health care for adults with ID, as physicians try their best, but may inadvertently harbor prejudices and/or be unsure as to how to best care for patients with ID [33, 36].

In other fields, exposure to people with ID through one's personal life (i.e., family, volunteer work, neighborhood exposure, etc.) has increased comfort with this population [37]. Conversely, people who have not yet had the opportunity to get to know people with ID in nonclinical settings are more likely to express negative or outdated attitudes towards people with ID and their capabilities [36, 38, 39]. As the majority of primary care physicians lack exposure and education [36], we theorize that established barriers to effective health care for adults with ID are intensified in the arena of sexual health, a sensitive topic for those with and without disabilities. This barrier is compounded by a lack of clear evidence regarding sexual health care for adults with intellectual disabilities [2].

### 2.2. Practice Recommendations

There is evidence suggesting that exposure to people with intellectual disabilities increases provider comfort in caring for this population [38, 39]. Providers are encouraged to seek education and opportunities for exposure to adults with ID and to incorporate ID into medical education. Empirically, we advise primary care providers to familiarize themselves with the barriers to care facing adults with ID and sensitively broach the topic of sexual health with all patients, including those with ID.

## 3. Sex Education

### 3.1. Background

Adults with ID may lack information about sexuality and sexual health [41–43] and often lack both formal and informal opportunities for learning about sexuality [42]. In one study, adults with ID were more likely than both adults with physical disabilities and the general population to state that they did not have all the sexual knowledge that they would like to have [44]. Additionally, adults with ID are more likely to get sexual information from questionable sources, such as television [18, 43], and to express misconceptions related to reproductive anatomy and physiology, sexuality and sexual health [18, 30, 41, 42, 45–47]. We hypothesize that this lack of biological and health knowledge may correlate to a lack of practical knowledge (i.e., how to properly put on a condom) that may put adults with ID at increased risk of negative sequelae of sexual activity.

There is a general lack of evidence regarding what constitutes effective sexuality education for adults with ID [43, 48]. Education about basic reproductive physiology, communication about sexuality and intimacy, gender differences, and safer sex has been theorized to increase the ability of women with ID to recognize and report abuses perpetrated against them [43, 49, 50]. It has been hypothesized that effective sex education for people with ID should include decision making skills, as adults with ID may have less opportunities to practice decision making than their peers without disabilities [49, 51] and should include practical and person-centered planning [52].

### 3.2. Practice Recommendations

Adults who lack sexual information may feel embarrassed discussing sexuality, so it is important to approach the topic sensitively and to create an environment in which it is safe to ask questions. If possible, it may be beneficial to schedule longer appointments in order to provide more thorough education, which enables informed decision making about sexual health care. It is also important to assess the patient's knowledge base when presenting options, in order to ensure comprehension.

Empirically, we encourage primary care providers to offer sex education when appropriate or feasible. It should be noted that family members may have strong opinions regarding sexuality and their loved one, and providers are encouraged to individualize care. It may be appropriate to include family members in patient education regarding sexuality. Conversely, an adult patient may prefer to discuss sexuality without family members or support workers present. In this case, physicians are encouraged to respect patient autonomy. It may be appropriate to ask family members to step out of the room in order to ask the patient whom she prefers to have present. It should be noted that patients who are their own legal guardians have the right to make medical decisions for themselves, including decisions about sexual health care. If a patient has a legal guardian, the guardian must consent to medical decisions, but it may still be appropriate to provide opportunities for private conversation during visits.

## 4. Sexual Abuse & Consensual Sexuality

### 4.1. Background

Though problems with tracking, reporting, and definition make it difficult to determine the exact prevalence, adults with intellectual disabilities are thought to be at high risk for abuse, including sexual abuse, with rates estimated as high as over half of all women with ID [50]. In one Australian study, almost 6% of police reports related to sexual assault involved an adult with ID, though these adults comprise just 0.8% of the Australian population [53]. This finding is especially significant considering research suggesting that women with ID are less likely than other women to report abuse [50, 54, 55] In addition, certain aspects of the disability experience, such as the need for paid personal caregivers, who are often alone with their clients, potentially increased dependency on family and support staff (creating a power differential that can create a barrier to reporting abuse) and decreased economic status can increase vulnerability to abuse [56]. As discussed above, sex education is thought to be beneficial in combating sexual exploitation, as it may increase participants' abilities to recognize and report abuse [43, 57].

It is important that primary care providers be alert to the potential for sexual abuse of their patients with ID. Conversely, it is also important to be aware that some adults with intellectual disabilities can and do engage in consensual sexual activity [58]. Capacity to consent is a legal definition that is not determined by medical providers, but it should be noted that just as many adults with ID are capable of providing informed consent in regard to their medical care, many adults with ID may be capable of making informed sexual decision, and of consenting to mutually desired sexual activity [59].

The self-advocacy movement has asserted that adults with ID have the same right to sexual expression as their peers without disabilities, and many regard sexuality as a human right [60]. This movement uses a rights-based framework that focuses on the human rights, rather than the limitations, of people with ID. This framework suggests that adults with ID be supported in accessing opportunities for consensual sexual expression if they desire to do so. Adults with ID may need support in multiple areas of their lives, including their sexuality, and may need assistance with sexual expression, such as help undressing before intimacy [51, 52]. However, direct support workers who assist people with ID often lack training in supporting positive sexuality or even providing basic information to their clients [51]. An example of a fact sheet supporting the sexual rights of adults with ID provided by the NSW Council for Intellectual Disability, an Australian advocacy organization, can be accessed at http://www.nswcid.org.au/health/se-health-pages/sexuality.html.

### 4.2. Practice Recommendations

The tension between protecting adults with ID from abuse while also respecting their right to consensual sexual expression may at times be difficult for providers to navigate. Empirically, it is important to screen patients with ID for abuse without assuming that all sexual activity is categorically abusive. Adults with ID should be asked if they are sexually active, with a definition of this term provided in accessible language, if necessary. Providers should then attempt to ascertain if sexual activity was/is consensual. It may be beneficial to ask accompanying staff or family members to sit out for this part of the visit to enable truthful disclosure. If abuse is discovered, US medical personnel of all types are mandatory reporters [61]. Exact reporting requirements and procedures will vary nationally and across states/provinces, but many states and countries mandate that even the suspicion of abuse must be reported. Some governments provide protection for reporters, including anonymous reporting options and immunity from prosecution [61]. However, it should be noted that response and enforcement can also vary [62], and providers are encouraged to research the requirements, protections, policies, and enforcement strategies of their jurisdiction. The Disability and Abuse Project can provide additional resources at http://www.disability-abuse.com/.

## 5. Contraception

### 5.1. Background

Contraception use remains a controversial topic for adults with intellectual disabilities. Women with ID are more likely than other women to use contraception and to request hysterectomy, in order to manage menstruation, including menstrual hygiene [40]. Research has shown that the gynecological health needs of women with ID are less likely to be met [45, 63], though women with ID suffer from premenstrual syndrome and other menstrual disorders at similar rates as the general population [40].

Other women with ID may desire contraception in order to protect against pregnancy. Women with ID face barriers to obtaining appropriate contraception. Very few women with ID are routinely asked about their contraceptive needs by their primary care providers [45], and family planning clinics may not be accessible or inclusive [64]. Family members may fear that contraceptive use could mask abuse by preventing pregnancies that might alert caregivers to rape [59] and so might discourage contraception. Residential setting may also play a role, according to a study of Belgium's health databases, which found that women who lived in nonfamilial residential settings were more likely to use contraception if their residential setting required it and/or permitted sexual intercourse [65]. In addition, barriers related to disability may make finding an appropriate contraceptive method more difficult, as women might need reminders to take oral contraceptives regularly or might be on medications that contraindicate hormonal methods [45, 59].

Though a full discussion of sterilization's legal and ethical implications is beyond the scope of this paper, it is important to recognize the long history of compulsory and involuntary sterilization of people with ID in the United States [24], Australia, and other nations. In the USA, the Eugenics movement influenced a trend towards compulsory sterilization (defined as sterilization of a population group for societal purposes, rather than personal purposes of the individual) of women with ID [66]. Compulsory and involuntary sterilization (defined as sterilization of a person who is unable to provide consent) of institutionalized individuals with ID was legally legitimized in 1927 in the case of Buck versus Bell. Though an official apology was issued 75 years later in regard to this case, and laws mandating compulsory and or involuntary sterilization have been repealed, sterilization of a person who is incapable of consenting to the procedure is still legally permissible in the USA if benefit to the individual can be established [66]. The United Nations Human Rights Commission recommends against sterilization of girls with disabilities, and recommends that nations develop systems of protection [67]. The Australian Senate is currently considering legislation that would ban the involuntary sterilization of minors with ID, as well as providing additional oversight [68]. Though sterilization may be the appropriate option for some people with ID in treating medical conditions, it should be considered a last resort, used only if less invasive options have been exhausted [44, 45, 58].

### 5.2. Practice Recommendations

**Figure 1:**
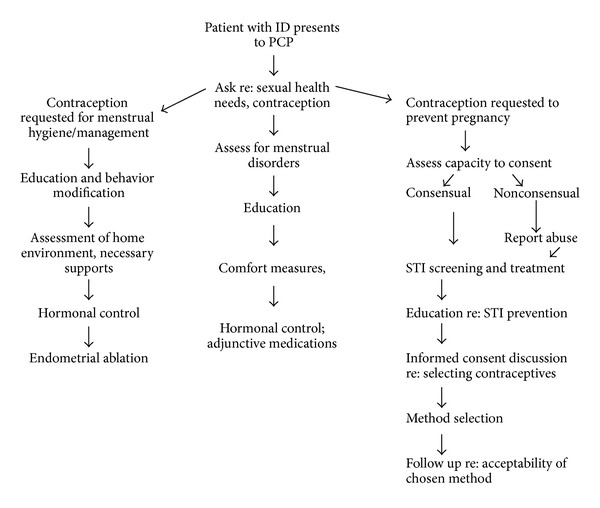
Algorithm for contraceptive decision making, updated from Paransky and Zurawin [40].

We theorize that all patients, including women with ID, should be asked about their contraceptive and gynecological needs, including any issues with menstrual regularity and pain. Treatment options, including comfort measures and complimentary therapies as appropriate should be provided for any issues related to menstrual health. When a woman with ID requests contraception, it is important to clarify the reasoning behind the request. Since many women with ID can be successfully taught to manage menstrual hygiene, not all authorities consider menstrual management to be an appropriate indication for contraception use [58, 69]. Recognizing women with ID are more likely than the general population to use contraception for reasons other than the prevention of pregnancy; Paransky and Zurawin have developed a decision tree model for use by health care provides when a patient with ID and/or her caregivers request contraception or sterilization [40]. We have updated their algorithm to reflect a rights-based framework. Please note that primary care physicians should of course consider individual risk factors when applying our adapted algorithm, found in Figure 1.

We theorize that clinicians should also remain open to providing contraception to women with ID in order to prevent pregnancy. It is important to individualize care for these patients and should work with the patient to determine the most appropriate method. Clinicians can help women assemble support for using the chosen contraception method successfully (i.e., identifying a support worker or system to help remind the woman to take her medication, etc.) In addition, though a thorough discussion of legalities surrounding sterilization is beyond the scope of this paper, primary care providers are encouraged to familiarize themselves with the relevant laws and policies of their locality.

## 6. Screening Tests: STI and Cervical Cancer Screenings

### 6.1. Background

The importance of regular cervical cancer screening (Pap testing) for women in the general population is well understood. However, controversy exists regarding cervical cancer screening for women with ID due to the invasive nature of the test and the low rate of cervical cancers in women who are sexually inactive. Barriers to regular Pap screening include difficulty with communication and patient cooperation as well as physical disabilities that may make the procedure more difficult [63, 70]. In addition, some patients may find the speculum exam traumatic [70], a situation that has the potential to jeopardize the physician-patient relationship, decrease patient cooperation, and/or increase patient anxiety regarding medical care. In light of this, it is not surprising that women with intellectual disabilities have very low rates of cervical cancer screenings [71], though it has been noted that accurate estimates of screening prevalence are difficult to ascertain in this population [72]. Rates of abnormal Pap test are also low among women with ID [73], probably due to a much lower incidence of sexual activity and therefore a much lower incidence of HPV.

There is less evidence in regard to sexually transmitted infection (STI) testing related to adults with intellectual disabilities. Little is known regarding rates of STIs in this population, though adults with ID are less likely than other adults to be tested for HIV [74] and to have lower levels of knowledge regarding STI and HIV prevention [75]. One study found excess risk of STI among female adolescents with ID [76]. As discussed, women with ID are less likely to disclose sexual activity and are more likely to be victims of sexual abuse, leading to a potentially increased risk of STIs.

### 6.2. Practice Recommendations

Though there are clear benefits to cervical cancer screening in the general population, the evidence is less clear related to women with ID. We think that providers should individualize care by determining whether the patient is sexually active in order to make a decision about Pap testing. (This determination should take into account the increased prevalence of abuse and assault among women with ID.) Additionally, there is some evidence to suggest that obtaining a Pap specimen using a “blind” technique and liquid cytology may be less traumatic for patients than a conventional speculum exam. This technique involves inserting only one finger into the vagina, manually locating the cervical os and guiding a cytobrush across the os. This method produces a lower than usual rate of specimen adequacy (as defined by presence of endocervical cells) yet may be a better alternative than foregoing the Pap test completely [77]. We recommend routine screening women with ID for STIs as a routine aspect of primary care, due to the increased prevalence and underreporting of abuse in this population and the highly treatable nature of most STIs. STI testing can be completed using urine or blood samples, as opposed to vaginal cultures, in order to increase acceptability [78].

## 7. Pregnancy and Parenting

### 7.1. Background

It is important that primary care providers recognize that pregnancy is possible for most women with ID and may be desired by some. (Some genetic and other syndromes, such as Fragile X Syndrome, may cause sterility [63]; however, the vast majority of people with ID have unspecified ID [79].) Due to the lack of a national tracking system or database in the USA, it is impossible to know exactly how many women with ID become pregnant or give birth each year. Data from Holland's national health database suggests that 1.5% of adults with ID are parents [80] and similar, though slightly lower rates were found in Germany [81]. It is interesting to note that Dutch policy favors a rights-based framework which suggests that any adult who desire it has the right to plan a pregnancy [80], and focuses on providing support for successful parenting. It is unclear whether this policy framework might encourage higher rates of parenting among people with ID than we would see in the USA and other locations. We also lack reliable data regarding the percentage of planned versus unplanned pregnancies among women with ID, and we do not know how many pregnancies are the result of sexual assault. It is therefore vital to avoid making assumptions when a pregnancy is diagnosed in a woman with ID.

Women with ID who do become mothers face significant barriers and substantial discrimination, including what some describe as excessive and discriminatory child protective services (CPS) involvement [82]. Parents with ID who become involved with CPS are less likely to have prior court involvement and are much less likely to be charged with child abuse than other parents. Though they have a higher rate of compliance than other CPS involved parents, parents will ID are less likely to be offered supportive services, such as parenting classes, and are more likely to lose custody of their children [82]. Child protection policies may be outdated in terms of the rights of people with ID [82, 83]. With all parents, support plays an important role in parenting success; qualitative studies of parents in the USA and internationally found that support may be particularly vital for parents with ID, especially long-term supportive relationships [80–82, 84]. The Arc, the largest national association of and for persons with ID in the USA supports the right of people with ID to become parents. This group favors establishing the social services and supports needed to enable positive, successful parenting by adults with ID who choose to have children [83]. However, there is a documented “support gap” for parents with ID [85].

### 7.2. Practice Recommendations

While clinicians who suspect sexual assault have a moral (and often legal) duty to report, we suggest that clinicians carefully assess the capabilities and desires of a pregnant woman or mother with ID. Does she herself express a desire to parent? Was her pregnancy planned? Women with ID may choose to terminate a pregnancy, but, to the extent possible, this should be a fully informed and shared decision between the woman, her family, and her physician, with the woman's wishes respected. It may be appropriate to involve a social worker or other professional experienced in populations with ID in the decision making discussion. It should never be assumed that all women with ID who become pregnant should terminate. Likewise, while child abuse and neglect must be reported, primary care providers can act as important members of the support team needed to ensure successful parenting in women with ID who desire motherhood [83].

People with intellectual disabilities are a known disparity population, and sexual health care is a particularly neglected area of health care for adults with ID. Primary care providers can play an important role in addressing this disparity through the provision of sensitive and appropriate sexual health care.

## Supplementary Material

Supplementary Table 1. Summary of Evidence and Clinical Recommendations
