# Supplementary material for: Sexual and Reproductive Health Care for Women with Intellectual Disabilities: A Primary Care Perspective
Source: Int J Family Med. 2013 Dec 12;2013:642472. doi: 10.1155/2013/642472 (PMC3876698; doi:10.1155/2013/642472)
Supplement: Supplementary file 1 — Supplementary Table 1. Summary of Evidence and Clinical Recommendations [file 642472.f1.doc]

Table 1. Summary of Evidence and Clinical Recommendations:

| Issue | Summary | Recommendations |
| --- | --- | --- |
|  |  |  |
| Barriers to Care | Most adults with ID are cared for in the community; community based providers often lack education and experience in caring for this population.  Sexual health may be a particularly difficult topic for providers to broach with their patients with ID | Evidence suggests that exposure to this population increases comfort-educational interventions for health care providers regarding primary care of this population are important.  Health care providers should seek opportunities for exposure to adults with ID.  It is important to broach the topic of sexual health with all patients, including those with ID |
| Sex Education | Adults with ID lack access to sexual education and are more likely than the general population to lack information and to harbor misconceptions.  Lack of practical knowledge may place adults with ID at higher risk.  We lack evidence related to what constitutes effective sex education for this population. | Approach discussions of sexuality with sensitivity  Assess patient’s knowledge base and provide education as needed, using accessible language. Schedule longer appointments if possible.  Use a rights-based framework that accepts consensual sexual expression |
| Sexual Abuse and Consensual Sexuality | Adults with ID are at higher risk of sexual abuse than the general population; aspects of the disability experience make these adults particularly vulnerable.  Many adults with ID are capable of consenting to sexual activity; adults with ID have the same right to sexual expression as other adults. | Be alert to the potential for sexual abuse; screen all patients  Health care providers are mandatory abuse reporters  Avoid assuming that all sexual activity is abusive-ascertain whether reported sexual activity was consensual. |
| Contraception | Women with ID suffer from common menstrual disorders at similar rates as other women; contraception can be used to treat menstrual disorders  Women with ID lack access to contraceptives | Ask all women with ID about menstrual regularity and pain  Ask women with ID about their contraceptive needs  Individualize care to help women beat the barriers to appropriate contraception  Consider sterilization only as a last resort when all other options have been exhausted |
| STI and Cervical Cancer Screening (Pap Testing) | Women with ID have lower rates of cervical cancer and STI screening than other women.  Providers may hesitate to perform these tests, or may incorrectly assume that a women with ID is not sexually active.  Women with ID have lower rates of abnormal Pap smears, probably due to lower rates of sexual activity. Little is known about rates of STIs in this population, though women with ID are at higher risk of experiencing sexual abuse | Provide opportunities for informed decision making related to cervical cancer screening. A modified technique may be used to obtain a specimen for Pap testing if a speculum exam is not feasible or tolerable.  Don’t assume a lack of sexual activity in patients with ID.  Offer all patients with ID STI screening as a routine part of primary care. |
| Pregnancy and Parenting | Pregnancy is possible for most women with ID, and desired by some  The U.S. and other countries lack data regarding rates of pregnancy and parenting among women with ID; 1.5% of Dutch adults with ID are parents  Parents with ID face important barriers, including discrimination, lack of services | Provide preconception care to women with ID  Provide individualized care and options counseling (with support from social work and other professionals as appropriate) if a patient presents with a pregnancy; provide opportunities for shared decision making  Refer pregnant/parenting patients with ID to support services as needed. |
|  |  |  |
